# Physical activity and diet behaviour in colorectal cancer patients receiving chemotherapy: associations with quality of life

**DOI:** 10.1186/1471-230X-9-60

**Published:** 2009-07-27

**Authors:** Lynette E Stephenson, D Gwyn Bebb, Raylene A Reimer, S Nicole Culos-Reed

**Affiliations:** 1 Faculty of Kinesiology, University of Calgary, 2500 University Dr. NW, Calgary Alberta, T2N 1N4, Canada; 2 Department of Psychosocial Resources, Tom Baker Cancer Centre, Holy Cross Site, 2202 2nd St. SW, Calgary, Alberta, T2S 3C1, Canada; 3 Department of Oncology, Tom Baker Cancer Centre/Faculty of Medicine, 1331 29th St. NW, Calgary, Alberta, T2N 4N2, Canada; 4 Department of Biochemistry and Molecular Biology, Faculty of Medicine, 3330 Hospital Drive NW, Calgary, Alberta, T2N 4N1, Canada

## Abstract

**Background:**

The relationship between colorectal cancer (CRC) risk and physical activity and dietary habits has been well-established, but less is known about the relationship between these behaviours and quality of life (QOL) post-diagnosis. Moreover, it is unknown whether this relationship is consistent across cancer stage or treatment setting. Thus, the purpose of this study was to assess current diet and physical activity behaviour in CRC survivors receiving systemic chemotherapy, and to examine potential associations between these behaviours and quality of life. A secondary purpose was to examine the association between social support, diet, and physical activity behaviour in this population.

**Methods:**

Using a cross-sectional survey, 67 CRC survivors currently receiving chemotherapy in Calgary, Alberta completed the survey package. Measures included demographic and medical data, physical activity levels, diet behaviour, QOL, and social support.

**Results:**

In a largely metastatic sample (63%), approximately half were meeting national dietary guidelines (58%), less were meeting national physical activity guidelines (26%), and a small number were meeting both (17%). However, only 12.3% (n = 8) reported completely sedentary behaviour, and 7 of these 8 participants were receiving metastatic treatment. Neither behaviour was significantly associated with QOL or perceived social support. Furthermore, there were no significant QOL differences between those treated with palliative intent or adjuvant therapy. Important group differences emerged between those meeting and not meeting the guidelines, and associations between QOL, age, BMI, and provisions of social support.

**Conclusion:**

These findings provide insight into lifestyle behaviours of CRC survivors currently receiving systemic chemotherapy, and the differences in perceived QOL as affected by severity of disease and treatment setting. Prospective studies in a larger sample of CRC survivors on chemotherapy are needed to confirm lifestyle behaviour patterns and identify factors related to QOL that are unique to this population, especially during metastatic treatment.

## Background

Colorectal cancer (CRC) is the second most prevalent cancer in Canada and third in the United States for men and women combined [1,2]. With increased early detection and effective treatment, as well as an aging population, the number of CRC survivors is growing significantly. Although improvements in treatment regimens have beneficially impacted the prognosis of CRC, several quality of life (QOL) issues result from potential side effects of such aggressive treatment [3]. Consequently, shifting part of our focus in research and program development to address issues of QOL and survivorship has become essential [4].

Physical activity and diet are two lifestyle behaviours shown to significantly impact both QOL and survivorship in cancer populations [5]. Within the CRC population specifically, physical activity and diet behaviour have shown both an independent and combined positive association with QOL [6-10]. Specifically, sufficient levels of physical activity (i.e., meeting published guidelines) or consuming at least five servings of fruits and vegetables per day (i.e., 5-A-Day recommendation) have been associated with significantly higher measures of self-reported QOL. Additionally, health behaviour clusters have been identified (i.e., meeting guidelines for multiple behaviours such as diet, physical activity, and not smoking) which appear to have a cumulative impact on QOL [6,7]. It has therefore been suggested that targeting multiple behaviours may prove more effective in enhancing QOL than focusing on individual behaviour change in the cancer survivor population [5].

However, not all studies concur with this impression. One recent study in particular found that no lifestyle variable was a significant predictor of domain-specific or overall QOL after CRC [11]. This discrepancy may be due, in part, to the varying QOL and health behaviour measures used, as well as differing sample characteristics. However, more research is warranted to determine whether the relationship between lifestyle and QOL persists across stages of CRC, treatment type (i.e., surgery, chemotherapy, radiation), or setting (i.e., adjuvant or metastatic). For example, physical activity trials during treatment in the general cancer literature have shown mixed results on impacting QOL. Findings have ranged from negative trends on QOL subscales, to positive statistically nonsignificant trends, to moderately significant beneficial effects (i.e, mental health, role and emotional functioning) [12-16].

Furthermore, despite the established link between lifestyle and CRC, several reports indicate that adherence to suggested guidelines for either behaviour is low. For example, recent studies have reported 25 to 54% of CRC survivors meeting varied published activity guidelines [7-10,17], and 16 to 47% achieving the 5-A-Day diet recommendations [7,8]. However, it is unknown whether the variance in adherence to both behaviours is consistent across all stages of CRC, or treatment setting. A greater understanding of factors underlying adherence to either behaviour would provide important insight into whether current guidelines are appropriate for all CRC survivors regardless of stage or treatment, and in turn guide future behavioural interventions in this population. Since these behaviours are linked to decreased recurrence risk and increased survival rates in CRC [18-20], as well as improved QOL [7], promotion of healthy lifestyle changes in this population is imperative.

The purpose of this study was to assess current physical activity and diet behaviour in CRC survivors undergoing chemotherapy, and examine how these behaviours may be related to QOL. Based on previous research in CRC, we hypothesized that adherence to either behaviour guideline would be low. However, we did predict that meeting physical activity and diet guidelines would correspond with higher self-reported QOL scores. A secondary purpose was to examine the association between social support, diet, and physical activity behaviour in this population. It has been suggested that social support may be a key factor in explaining some of the variance in healthy behaviours [21-23]. It was hypothesized that those with greater provisions of social support would report greater adherence to both behaviour guidelines.

## Methods

This study was a cross-sectional survey design using a convenience sample of CRC patients currently receiving chemotherapy. Participants included CRC patients of the gastrointestinal (GI) tumour group at the Tom Baker Cancer Centre (Calgary, AB, Canada). Upon ethical approval from the Conjoint Health Research Ethics Board, University of Calgary, participants were recruited on-site during clinic visits in which the medical oncologist first obtained patient permission to be approached by the researcher. Eligibility criteria included: a) primary diagnosis of colon or rectal cancer, b) receiving chemotherapy (adjuvant or metastatic) not concurrent with radiotherapy, c) at least 18 years of age, and d) ability to read and write in English. By definition, adjuvant therapy is given with curative intent after the resection of all detectable disease, whereas metastatic treatment is given with palliative intent when the disease has spread to other parts of the body and is no longer modifiable. Of note, patients receiving radiotherapy were excluded based on the nutritional implications associated with this treatment regimen. A total of 112 patients were eligible for the study over a 6-month period, and 108 consented to complete the survey package and receive one reminder phone call to return the completed package.

### Measures

The survey package was comprised of demographic and medical questions, physical activity and diet behaviour assessments, QOL and social support. Medical data was obtained through patient records to ensure accuracy. Specific measures included the following.

#### Physical Activity Behaviour

Physical activity was assessed using Godin's leisure score index (LSI) of the GLTEQ (Godin Leisure Time Exercise Questionnaire) [24]. The LSI is a 3-item measure assessing the frequency and duration of mild, moderate and strenuous bouts of physical activity performed during free time in a typical week. In this study, participants were asked to recall their physical activity levels over the past month. The LSI was then computed into MET hours, which included only moderate and strenuous activity in the present study to reflect the Canadian guidelines outlined in the questionnaire: "At least 30 minutes of moderate activity 4 or more times per week" [25]. Thus, participants needed to perform at least 10 MET hours per week to meet the guideline. The LSI has been successfully used with adult cancer patients and survivors [26,27], and an independent evaluation of its reliability and validity compares favourably to nine other self-report measures of exercise [28].

#### Diet Behaviour

Diet was assessed using a 3-day diet record. This dietary measure is said to be the most accurate for mean macronutrient content and appropriate for use in studies where subjects may consume a wide variety of foods [29]. Participants were instructed to record their daily consumption over a period of three days, one of which must be a weekend day. From the diet records, percent of total recommended daily energy and macronutrient intake (i.e., fat, carbohydrates, and protein) were assessed based on individual height, weight, age, and gender. Servings of fruit and vegetables were also assessed. Participants were then classified as meeting or not meeting Canadian diet recommendations based on meeting the appropriate range for each macronutrient, the appropriate energy intake, and fruit and vegetable consumption. Specifically, participants had to be within the acceptable daily macronutrient distribution ranges for adults: carbohydrates = 45–65% of total energy intake, proteins = 10–35% of total energy intake, and fats = 20–35% of total energy intake [30]. These ranges reflect recommended daily servings in Canada's Food Guide [31].

#### Quality of Life

QOL was assessed using the Functional Assessment of Cancer Therapy – Colorectal module (FACT-C). This tool contains five subscales including physical, social-family, emotional, functional, and a CRC specific concerns subscale. It has been shown to be reliable and valid for assessing QOL within the CRC population [32,33].

#### Social Support

Perceived social support was assessed using the Social Provisions Scale (SPS), which provides both a global social support score, and six relational provisions: attachment, social integration, reassurance of worth, reliable alliance, guidance, and opportunity to nurture [34]. Scores from this instrument have been shown to be reliable and valid within both healthy and cancer populations [22,35].

### Analysis

The raw data was analysed using SPSS Version 15.0. The 3-day diet records were first analysed by a registered Dietician using Diet Analysis Plus software (Thomson, Wadsworth, Toronto, ON) to provide total daily energy intake, and macro and micro-nutrient intake for each participant. Reports were specific to participants' age, gender, self-reported height, and weight. Percentage of recommended daily amounts and macronutrient intake was then added to the SPSS database for statistical analyses. After data input and cleaning, the normality of the data was analyzed (i.e., skewness and kurtosis). The data was within the requirements of normal distribution, thus no data transformations were necessary. Descriptive statistics included means, ranges and standard deviations on all demographics, medical variables, physical activity and diet behaviour, QOL, and social support. Individual t-tests and chi-squares were performed as appropriate to test differences between those meeting and not meeting the guidelines for both behaviours. Correlations and analyses of variance examined the relationship between behaviour, social support and QOL.

## Results

Of the 112 eligible participants seen in clinic over a six-month period, 108 (96%) consented to participate in the study. A total of 67 packages were returned (response rate = 62%), of which 63 were completed in full (i.e., both the questionnaire and 3-day diet record were returned). There were no statistically significant differences in medical data between those who completed and those who did not complete the survey. Sociodemographic data was only available for those who completed and returned the survey package.

### Sample Characteristics

Complete demographics and medical information for the study sample (n = 67) is presented in Tables 1 and 2. In summary, the sample was reflective of national statistics for CRC. Participants' ages ranged from 29 to 84 years (M = 60.4), 52.2% were male, the majority were Caucasian (90.8%), married or common law (81.5%), many had a university or college education (35.9%), and 30.5% had an annual income greater or equal to $80,000. The medical data shows the mean time since diagnosis for the entire sample was 13.9 months (SD = 14.1). A small number of participants had an ostomy appliance (n = 15), and all participants were currently receiving chemotherapy with a mean time on active treatment of 5.9 months (SD = 7.7). More than two thirds were being treated for metastatic disease (62.7%), and the majority were initial diagnoses receiving Capecitabine in either treatment setting.

**Table 1:** Participant demographics

Characteristic	N	Percent
Age (M, *SD*)	60.4, *13.2*	
Gender		
Male	35	52.2
Female	32	47.8
Race (n = 65)		
Caucasian	59	90.8
Asian	5	7.7
Black	-	-
Hispanic	-	-
Aboriginal/Métis	1	1.5
Other	-	-
Marital Status (n = 65)		
Married/Common law	53	81.5
Divorced/Separated	5	7.7
Widowed	5	7.7
Never Married	2	3.1
Education Level (n = 64)		
Some high school	10	15.6
Completed high school	12	18.8
Some university/college	12	18.8
Completed university/college	23	35.9
Some or completed grad school	7	10.9
Annual Income (n = 59)		
<20,000	4	6.8
20,000–39,999	14	23.7
40,000–59,999	17	28.8
60,000–79,999	6	10.2
>80,000	18	30.5

**Table 2:** Participant medical characteristics

Characteristic	N	Percent
Months since diagnosis (M, *SD*)	13.9, *14.1*	
Months on treatment (M, *SD*)	5.9, *7.7*	
Ostomy Appliance (n = 67)	15	23.4
Adjuvant Therapy	25	37.3
Colon Stage II	3	4.5
Colon Stage III	20	29.9
Rectal Stage III	2	2.9
		
Initial diagnosis	21	31.3
Recurrence	4	6.0
		
Capecitabine	12	17.9
Folfiri	1	1.5
Folfox	9	13.4
Fufa	3	4.5
		
Metastatic Therapy	42	62.7
Colon Stage IV	39	58.2
Rectal Stage IV	3	4.5
		
Initial diagnosis	26	38.8
Recurrence	16	23.9
		
Capecitabine	16	23.9
Folfiri	13	19.4
Folfox	10	14.9
Fufa	2	2.9
Tomudex	1	1.5

### Physical Activity and Diet Behaviour

Descriptive statistics for physical activity and diet behaviour can be seen in Table 3. For physical activity behaviour, participants (n = 65) had an average of 5.5 MET hours per week (SD = 9.9). With 10 or more MET hours needed to satisfy the public activity guidelines, only 26.2% (n = 17) of participants were currently meeting the minimum amount of weekly physical activity. Reviewing the average weekly duration and frequency of mild, moderate and strenuous activity, participants were performing mild activity most often (M = 32.2 minutes; M = 3.1 times/week), followed by moderate activity (M = 11.7 minutes; M = 1.1 times/week), and strenuous activity (M = 5.4 minutes; M = 0.3 times/week). Of interest, only 12.3% (n = 8) reported sedentary behaviour (i.e., zero minutes of activity for all three intensities), of which 87.5 (n = 7) were being treated with palliative intent.

**Table 3:** Physical Activity and Diet Behaviour

Behaviour	M	SD
Physical Activity (n = 65)		
Total activity duration (minutes)	49.2	51.5
Total activity frequency (times/week)	4.4	3.7
		
Mild activity duration (minutes)	32.2	36.3
Mild activity frequency (times/week)	3.1	2.8
		
Moderate activity duration (minutes)	11.7	20.4
Moderate activity frequency (times/week)	1.1	2.0
		
Strenuous activity duration (minutes)	5.4	18.6
Strenuous activity frequency (times/week)	0.3	1.2
		
MET hours per week*	5.5	9.9
		
Diet (n = 65)	%	SD
Average daily intake of recommended calories	106.3	31.1
		
Average daily calories from carbohydrate	50.6	7.8
Average daily calories from protein	17.3	4.2
Average daily calories from fat	31.3	6.3
		
Average daily recommended servings of fruits and vegetables	85.2	57.9

Meeting Guidelines (n = 65)	N	%

Meeting physical activity guidelines	17	26.2
Meeting dietary guidelines	38	58.5
Meeting guidelines for both behaviours	11	17.5

*MET hours include moderate and strenuous activity to reflect the minimum guidelines of activity stated within the questionnaire (At least 30 minutes of moderate activity 4 or more times per week, or at least 10 MET hours per week). MET hours was calculated by adding the outcome of strenuous frequency × strenuous duration × 9, with moderate frequency × moderate duration × 5, and dividing the sum by 60.

With regards to dietary behaviour, the majority of participants (58.5%) were meeting the national guidelines examined in this study. Looking at each macronutrient, participants fell within the acceptable range for carbohydrate (50.6%; acceptable range = 45–65%), protein (17.3%; acceptable range = 10–35%) and fat intake (31.3%; acceptable range = 20–35%). Furthermore, despite being on active treatment, on average participants were meeting more than their individual daily energy requirements (106.3%). Additionally, for comparison to previous studies assessing fruit and vegetable intake, 85.2% were achieving their recommended daily servings. Of note, only 17.5% of all participants met both physical activity and dietary guidelines.

### Quality of Life and Social Support

Social support and QOL scores can be viewed in Table 4. In summary, mean scores for the SPS were high for our sample and comparable to healthy population means [34]. Specifically, reliable alliance (assurance that others can be counted on in times of stress) was reported as the highest relational provision (M = 14.7, SD = 1.6), with opportunity for nurturance (providing assistance to others) being the lowest (M = 12.1, SD = 2.2). The FACT-C scores indicated relatively high overall QOL in our sample (M = 95.6, SD = 15.7). Social/family well-being was the highest reported QOL domain (M = 22.6, SD = 4.1), and physical well-being was the poorest domain (M = 16.7, SD = 6.1).

**Table 4:** Social Support and Quality of Life Descriptives

Measure	M	SD
Social Provisions Scale		
Total social support score (0–96; n = 64)	82.0	9.1
Subscale score (0–16)		
Attachment	14.2	1.9
Social integration	13.2	2.0
Reassurance of worth	13.8	1.9
Reliable alliance	14.7	1.6
Guidance	14.0	2.3
Opportunity for nurturance	12.1	2.2
		
FACT-C (quality of life)		
Total QOL score (0–136; n = 63)	95.9	15.7
Subscale score		
Physical well-being (0–28)	16.7	6.1
Social/family well-being (0–28)	22.6	4.1
Emotional well-being (0–24)	17.5	4.1
Functional well-being (0–28)	18.5	5.1
CRC-specific concerns (0–28)	20.5	4.2

### Behavioural Associations with Quality of Life

Bivariate correlations were performed between each behaviour and quality of life for the entire sample. The only significant findings were that the social/family well-being subscale was negatively correlated with meeting the physical activity guidelines (r = -0.301, p = 0.016), and the CRC-specific concerns subscale was negatively correlated with meeting the dietary guidelines (r = -0.255, p = 0.044). That is, higher scores on either QOL subscale correlated to the participants not meeting the corresponding behaviour guideline. Analyses of variance were used to further explore this relationship. Results from these tests confirm findings from our correlations, as significant between group differences on QOL measures were only found for physical activity and social/family well-being [F(1,62) = 6.20, p = 0.016], and diet and CRC-specific concerns [F(1,61) = 4.23, p = 0.044].

Given the limited associations between either behaviour and QOL, we also performed bivariate correlations to examine potential significant relationships with medical, demographic, or social support variables (see Table 5). The data revealed that BMI was negatively correlated with QOL (p = 0.033), and age (p = 0.001), total social support (p = 0.017), attachment (p = 0.010), social integration (p = 0.001), reliable alliance (p = 0.024), and guidance (p = 0.021) were positively associated with QOL. Of note, there were no significant differences in QOL scores for either treatment setting (i.e., adjuvant or metastatic).

**Table 5:** Correlates between QOL and significant demographic, medical, and social support variables

Variable	N	r	p
Age	63	0.409	.001
BMI	62	-0.271	.033
Total social support	63	0.299	.017
Attachment	63	0.321	.010
Social integration	63	0.410	.001
Reliable alliance	63	0.285	.024
Guidance	63	0.291	.021

### Group Differences

Individual t-tests were used to determine the significance between groups (i.e., those meeting or not meeting the stated guidelines) for continuous variables, and chi-square tests were used for categorical variables. Non-parametric Mann-U Whitney tests were also performed on ordinal measures and corresponded with t-test results; thus, t-tests have been reported. No significant differences were found in demographics, medical information or social support scores between those meeting and not meeting the guidelines for either behaviour.

## Discussion

The primary objective of this study was to assess current physical activity and diet behaviour in CRC survivors currently receiving systemic chemotherapy, and to examine associations between these behaviours and QOL. Of importance, the majority (63%) had significant burden of disease and were being treated with palliative intent. In light of these sample characteristics, the percentage of participants engaging in sufficient levels of activity and following the public dietary guidelines is impressive, although only 17% were meeting both behaviour guidelines.

Specifically, meeting dietary guidelines, broadly defined as meeting energy needs, appropriate macronutrient intakes, and fruit and vegetable intake, was better achieved in this sample than physical activity when benchmarked against current public health guidelines for both behaviours. Approximately half of the participants (58%) were achieving the appropriate range of daily calories from each macronutrient (i.e., carbohydrate, protein, and fat). On average, participants were also meeting or slightly exceeding their daily energy requirements (106.3%), which is consistent with intakes observed in breast cancer survivors [36]. Although previous reports indicate poor adherence to dietary recommendations in the CRC population, this has been based on assessing fruit and vegetable intake as part of the American Cancer Society's 5-A-Day campaign [7,8]. While direct comparisons cannot be made due to different diet assessment tools and sample characteristics, it should be noted that 85% of the current sample was meeting individually recommended servings of fruits and vegetables, which is considerably higher than previous reports. This may speak to the effectiveness of diet-specific health promotion strategies in the cancer population at large.

The public physical activity guidelines used in the current study call for at least 30 minutes of moderate activity, four or more times per week. Few were meeting these guidelines (26%), although this is comparable to other Canadian cancer survivors [37]. In comparison, a large number of our sample reported weekly mild activity (duration: M = 32.2 minutes; frequency: M = 3.1 times per week), and few (12%) reported no activity at all. This is positive, given the known risks of sedentary behaviour for survivors includes the development of comorbid conditions and cancer recurrence. Additionally, with the known potential side-effects from such aggressive treatment (i.e., neuropathy, hand and foot syndrome, diarrhoea, and nausea), achieving even mild activity (i.e., below the public activity guidelines) may be a more realistic goal for those with significant burden of disease undergoing treatment, and may still provide some benefits reported in the general cancer literature [38,39]. For example, preliminary physical activity and palliation research found that a 50-minute physical activity intervention twice a week for six weeks was feasible in a sample of 34 palliative cancer patients, in which nine were receiving chemotherapy [40]. Specifically, circuit training with strength, aerobic, and flexibility components was successful for improving fitness measures, symptom management (fatigue), and well-being. Of importance, intensity of exercise activity was not specified to the participants.

It is not unexpected that activity levels would be below recommended levels in this population. Peddle at al. found that only 9% of a large sample of CRC survivors (60% Stage III or IV disease) reported sufficient activity levels during treatment, which increased to only 25% post-adjuvant therapy [10]. Similarly, recent American and Australian studies have reported that only 32–54% of CRC survivors meet physical activity guidelines (both during and post-treatment survivor samples) [7-9,17]. While these studies used slightly different guidelines for minimum activity levels (i.e., >150 minutes of moderate activity per week) than the present study, it is apparent that activity levels are consistently reported as insufficient in CRC survivors. As there is a consistent call for establishing specific dose-response relationships (i.e., per different cancer type, stage, and treatment status) in the physical activity and cancer literature, future research should determine if lower levels of activity than the current guidelines suggest will still provide clinically meaningful health benefits for CRC survivors on chemotherapy, especially those with metastatic disease. For example, Spence et al. have reported their protocol for examining the health benefits of a supervised exercise program for CRC survivors immediately following adjuvant chemotherapy [41]. Such interventions are valuable for determining pertinent differences in achievable activity goals at specific stages of the cancer continuum and treatment settings.

An unexpected finding and contrary to our hypothesis, was that neither diet nor physical activity behaviour was significantly related to global QOL. Physical activity was negatively correlated to the social/family well-being subscale, and diet was negatively correlated to the CRC-specific concerns subscale (i.e., treatment-related side effects). Engaging in physical activity may be seen as taking away from important social/family interaction and thereby contribute to the observed negative association between activity and this subscale. The negative correlation between disease-specific concerns and following the dietary guidelines is not illogical, however, as the majority of the items on this subscale are directly related to the digestive process and those experiencing more symptoms are likely to adjust their diet to counter this.

The lack of associations between diet, physical activity and QOL may be due to the small sample of those actually meeting the physical activity guidelines (n = 17), dietary guidelines (n = 38), or both (n = 11), thereby making it difficult to detect significant relationships. Another potential explanation for this may be that 63% of the current sample was receiving chemotherapy for metastatic disease. Thus, this poor prognosis may significantly impact factors associated with QOL. As this was the first study to examine the association between lifestyle behaviours and QOL in a largely metastatic sample receiving systemic chemotherapy, these findings are important and need to be replicated.

With regards to QOL levels in the current study, both global and domain-specific QOL scores were lower than those reported in two previous CRC studies using the same measure. Specifically, the current sample reported a mean global QOL score of 95.9 (out of 136), compared to the means of 111.5 and 110 in these two previous studies [9,10]. Interestingly, the physical well-being domain score was the lowest of the five subscales in the present sample (M = 16.7; out of 28), but highest in the two other studies (M = 23.6 and 24). The lower QOL scores in the present sample likely reflect that the participants were undergoing adjuvant or metastatic treatment. The other two studies included CRC survivors both on and off treatment, and previous studies have reported that global QOL levels tend to increase over time (i.e., after treatment completion) in this population [42]. Understanding the specific domains of QOL most affected in CRC survivors, which appears to vary according to severity of disease and treatment setting, is valuable for tailoring future interventions targeting QOL outcomes.

In a further attempt to understand QOL in the current sample, exploratory analyses examined significant relationships between QOL and demographic, medical, and social support variables. It appears in this sample that BMI, age, and social support provisions have stronger correlations with perceived QOL than do the lifestyle behaviours. Specifically, lower BMI, older age, and greater provisions of attachment, social integration, guidance, and reliable alliance were significantly associated with overall QOL. Interestingly, no significant differences on any QOL scales were found between those treated in the adjuvant or metastatic setting. Contrary to what one might expect, this may imply that QOL is no worse when treated with palliative intent as compared to adjuvant therapy in this population. This needs to be confirmed in future studies.

With respect to age, Arndt et al. also found that younger CRC survivors reported worse QOL in functioning and symptom scales than older survivors [42]. It has been hypothesized from breast cancer studies that younger survivors may have less coping strategies and view their cancer as a greater threat to their lives [43-45]. The BMI and QOL correlation is also consistent with past research, which noted that those with lower BMIs reported higher perceived QOL [46]. A larger sample may replicate these findings, but also suggests that future studies with QOL as the primary endpoint may need to tailor interventions to age-specific needs of the participants.

The relationship between QOL and social support provisions found in this study is supportive of the findings in previous QOL and CRC research. Dunn and colleagues found that participants expressed emotional, instrumental, and spiritual support as most beneficial to their cancer experience [47], and Sultan et al. found that provision of emotional and instrumental support corresponded with improved QOL in survivors [48]. In addition, the recent work of Steginga et al. found social support to be significantly associated with all domains of QOL, and a significant independent predictor of social well-being, functional well-being, and overall QOL [11]. Although varying measures of social support were used in these studies, the culmination of these findings suggest that social support may be more directly related to subjective well-being for CRC survivors, and a key target for future QOL research in this population. However, as stated previously, knowing if this relationship is consistent along the cancer continuum or varies with stage and treatment of CRC needs to be investigated.

Although the present study was the first to look at both physical activity and diet behaviour exclusively in CRC survivors receiving chemotherapy, and the association between these behaviours and QOL, there are limitations to be noted. Primarily, the small and convenience sample limits the generalizability of the findings and ability to detect statistically meaningful relationships. Furthermore, the self-report nature of behaviour and the potential for social desirability may have influenced reporting for either behaviour. Using objective measures of behaviour in a larger sample to replicate these findings is important for providing the baseline data to effectively design future lifestyle trials in this population.

## Conclusion

In summary, this study found novel and important baseline information on current dietary and physical activity behaviour in the CRC population undergoing systemic chemotherapy. In light of our sample characteristics, especially the significant burden of disease in the majority of participants, the number who are engaging in physical activity is positive, and the high percentage following healthy food guidelines even more so. Moreover, the largely metastatic sample presents an understudied and important area for future research. Having these baseline measures are essential for future lifestyle research initiatives in this population, which are likely to vary with cancer stage and the different phases of the cancer experience. As well, our findings provide insight into the differences in perceived QOL as affected by treatment setting and severity of disease, compared with existing literature reporting QOL post-treatment and into survivorship in samples with less advanced disease. Evidence-based interventions are warranted in this population, especially to determine appropriate dose-response relationships between activity or diet and meaningful health benefits for survivors being treated with adjuvant or metastatic chemotherapy. Furthermore, the role of social support and other potential variables more highly associated with QOL should be explored to effectively target QOL outcomes specific to diagnosis and treatment.

## Competing interests

The authors declare that they have no competing interests.

## Authors' contributions

LES formulated the aims and hypothesis of the study, conducted the research, performed all statistical analysis and drafted the manuscript. SNCR was the formal MSc supervisor of LES, and contributed to and advised all aspects of the study. DGB was a co-investigator on the study and contributed to the research design, data collection, and critical revision of the manuscript. RAR was on the formal supervisory committee of LES, and contributed to interpretation of the findings and critical revision of the manuscript. All authors read and approved the final manuscript.

## Pre-publication history

The pre-publication history for this paper can be accessed here:

http://www.biomedcentral.com/1471-230X/9/60/prepub
